# How Do mAbs Make Use of Complement to Kill Cancer Cells? The Role of Ca^2+^

**DOI:** 10.3390/antib9030045

**Published:** 2020-09-04

**Authors:** Ronald P. Taylor, Margaret A. Lindorfer

**Affiliations:** Department of Biochemistry and Molecular Genetics, University of Virginia School of Medicine, Charlottesville, VA 22908, USA; mal9e@virginia.edu

**Keywords:** complement, therapeutic monoclonal antibodies (mAbs), Ca^2+^, fluorescence microscopy

## Abstract

We examined the kinetics and mechanisms by which monoclonal antibodies (mAbs) utilize complement to rapidly kill targeted cancer cells. Based on results from flow cytometry, confocal microscopy and high-resolution digital imaging experiments, the general patterns which have emerged reveal cytotoxic activities mediated by substantial and lethal Ca^2+^ fluxes. The Ca^2+^ fluxes are common to the reported pathways that have been utilized by other toxins in killing nucleated cells. These reactions terminate in very high levels of cell killing, and based on these considerations, we suggest additional strategies to further enhance mAb-based targeting of cancer with complement.

## 1. Introduction

Complement was first described and characterized by Bordet more than 100 years ago [1]; he demonstrated it to be a heat-labile factor in serum that promoted destruction (lysis) of bacteria and/or hemolysis of erythrocytes, each opsonized with the antibodies in immune sera. Complement is an important “first responder” that orchestrates the rapid clearance and destruction of a variety of microbial invaders as well as damaged and dying cells. It is therefore quite reasonable to anticipate that it should also be capable of destroying antibody-opsonized tumor cells. The importance of complement (C) in health and disease is now very well recognized and several outstanding reviews that describe its pathways and biological actions are available [2,3,4].

The first figure in the review in this volume by Golay and Taylor succinctly summarizes the important steps and controls in C-mediated killing of malignant cells opsonized with specific mAbs [5]. The traditional view of the mechanism by which C mediates the killing of antibody-opsonized cells was based on classic experiments that focused on C-mediated lysis of non-nucleated sheep erythrocytes that were first opsonized with polyclonal rabbit antibodies before they were brought into contact with a source of C and then incubated for a considerable period of time at 37 °C to promote hemolysis [6,7,8]. The results of these studies led to the concept that insertion of the membrane attack complex (MAC) pore(s) into the erythrocyte cell membrane allowed for influx of water and ions into the cell, ultimately leading to swelling of the cells followed by osmotic lysis and killing of the cells [6,7,8,9,10]. This model system has of course proven to be invaluable for dissecting out and identifying virtually all of the key components of C, including pathways, activating factors and inhibitors.

## 2. Nucleated Cells Are More Complicated: Important Questions

However, a considerable body of evidence, based on a series of studies by Shin’s group on the lysis of nucleated Ehrlich ascites cells (EACs) opsonized with rabbit polyclonal antibodies, suggested that the osmotic lysis concept could not explain how these nucleated cells were killed. Instead, the influx of Ca^2+^ mediated by MAC pores appeared to be the predominant lethal event [10,11,12,13,14,15]. The focus of these studies, completed more than 20 years ago, was on the terminal steps in the complement-dependent cytotoxicity (CDC) reaction. In the present review, in order to concentrate on mechanisms, we have examined multiple individual steps in the CDC reaction that start with mAb binding and end with cell death in a continuously monitored reaction mediated by Food and Drug Administration (FDA)-approved mAbs reacted with both cell lines and with primary malignant cells from patients with chronic lymphocytic leukemia (CLL) (Table 1).

In order to elucidate these mechanisms, we will address important questions with respect to the development of C-fixing mAbs to be used in cancer immunotherapy: How effective are these mAbs when they “attack” nucleated malignant cells in the presence of C (usually normal human serum (NHS))? What is the primary mechanism of cell killing? It would seem important to identify and optimize the primary killing mechanism to allow for efficient use of key resources, which comprise C and mAbs. Moreover, although the targeted malignant cells will employ a variety of defenses to ward off mAb-mediated attack [23,24,25,26,27,28,29,30], it is reasonable to ask that if the cells can be killed by *CDC*, then is there a common and general killing pathway? We developed several “complementary” (excuse the pun!) approaches to address these questions, based on quantitation and direct visualization and identification of many of the key separate steps in the CDC reaction. Many of our measurements of CDC made use of CD20 and CD37 mAbs in the killing of B cell lines and of CLL cells [16,17,18,19,20,21,22,31,32,33]. The similar patterns we have observed in these and other systems provide considerable evidence that there is indeed a common mechanism in the CDC reaction mediated by anti-tumor mAbs. In conducting these experiments, we followed the “ask-the-question” paradigm described by Nobel Prize investigator George Wald [34]:

“When it (scientific research) is going well, it is like a quiet conversation with Nature. One asks a question and gets an answer, then one asks the next question and gets the next answer. An experiment is a device to make Nature speak intelligibly. After that, one only has to listen.”

## 3. Experimental Strategies

Our approaches make use of fluorescently-labeled probes and fluorescent indicators, which include anti-tumor mAbs (lightly labeled with Alexa (Al) dyes, so as not to interfere with their activities, but adequate to monitor their binding to cells); C1q; mAbs 7C12 and 3E7 (specific for C3b/iC3b deposited on the cell) [35,36]; mAb HB43, specific for the Fc region of human IgG and able to recognize cell-bound mAbs [37]; Fluo-4, a Ca^2+^ indicator which is very sensitive to increases in the Ca^2+^ concentration in the cytoplasm and mitochondria of the cell [16]; tetramethylrhodamine methyl ester (TMRME), which is highly fluorescent only in viable mitochondria [16]; mAb aE11, specific for membrane attack complex (MAC)-associated C9 deposited on cells [38]; and vital fluorescent dyes such as propidium iodide (PI), 7-aminoactinomycin D (7AAD) and TO-PRO-3 which enter dead permeabilized cells and, upon staining nuclear DNA, become highly fluorescent, thus providing reliable markers for cell death and successful CDC [16,19,22]. We have been able to use this array of reagents to interrogate the quantitative and kinetic details of mAb-mediated killing of cancer cells in parallel experiments based on flow cytometry, high-resolution digital imaging in a flow cytometry environment (HRDI, Amnis technology [18]), and real-time multicolor confocal fluorescent microscopy movies.

## 4. Quantitation and Visualization of Early Steps in mAb-Mediated CDC: In Vitro and In Vivo Studies

**Figure 1:**
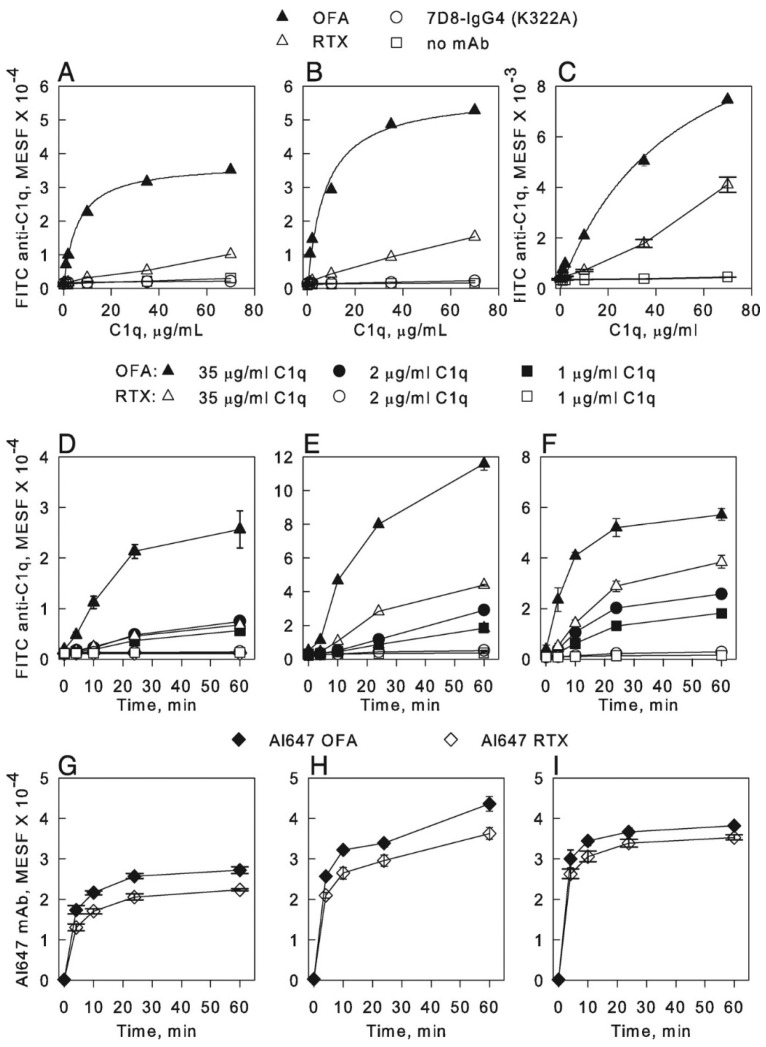
**OFA-opsonized B cells bind more C1q than RTX-opsonized B cells.** (**A**,**B**) Varying amounts of C1q were added to B cells suspended in complete RPMI 1640 medium and then reacted with either Al647 OFA, Al647 RTX, or Al647 7D8-IgG4 (K322A, does not bind C1q), all at 10 μg/mL; alternatively, no mAb was added. After incubation for 60 min at 37 °C, cells were washed and C1q binding determined by probing with FITC rabbit anti-C1q, followed by flow cytometry. (**A**), Raji cells. (**B**), Daudi cells. The results for binding of C1q to OFA-opsonized Raji and Daudi cells were fit to a binding isotherm, giving a *K*_D_ of 12 and 16 nM, respectively. All Al647 mAbs bound at high levels to the cells and mAb binding was the same in the presence and absence of C1q (not shown). (**C**), Binding of C1q to CLL cells opsonized with either OFA or with RTX, similar conditions and analyses (*K*_D_ for OFA-opsonized cells = 106 nM). (**D**–**I**), Cells were combined in cold medium with Al647 mAb and varying amounts of C1q and then incubated at 37 °C. Aliquots were removed at the indicated times, quenched with ice-cold BSA-PBS, washed, probed with FITC anti-C1q, and then analyzed by flow cytometry for C1q binding (**D**–**F**) and mAb binding by secondary probing with mAb HB43, specific for the Fc region of human IgG (**G**–**I**). (**D**,**G**), Raji cells. (**E**,**H**), Daudi cells. (**F**,**I**), Z138 cells. Results in all panels are representative of at least two similar experiments [17].

Table 1 summarizes the discrete events in mAb-mediated CDC that we have investigated. Initially, we were able to demonstrate rapid binding of the mAbs ofatumumab (OFA) and rituximab (RTX), specific for CD20, to B cell lines and to primary B cells from patients with chronic lymphocytic leukemia (CLL) [17]. We found that both RTX and OFA bound to the cells at the same levels (Figure 1, panels I–H). However, considerably more C1q was bound to the cells reacted with OFA (Figure 1, panels A–C) [17].

**Figure 2:**
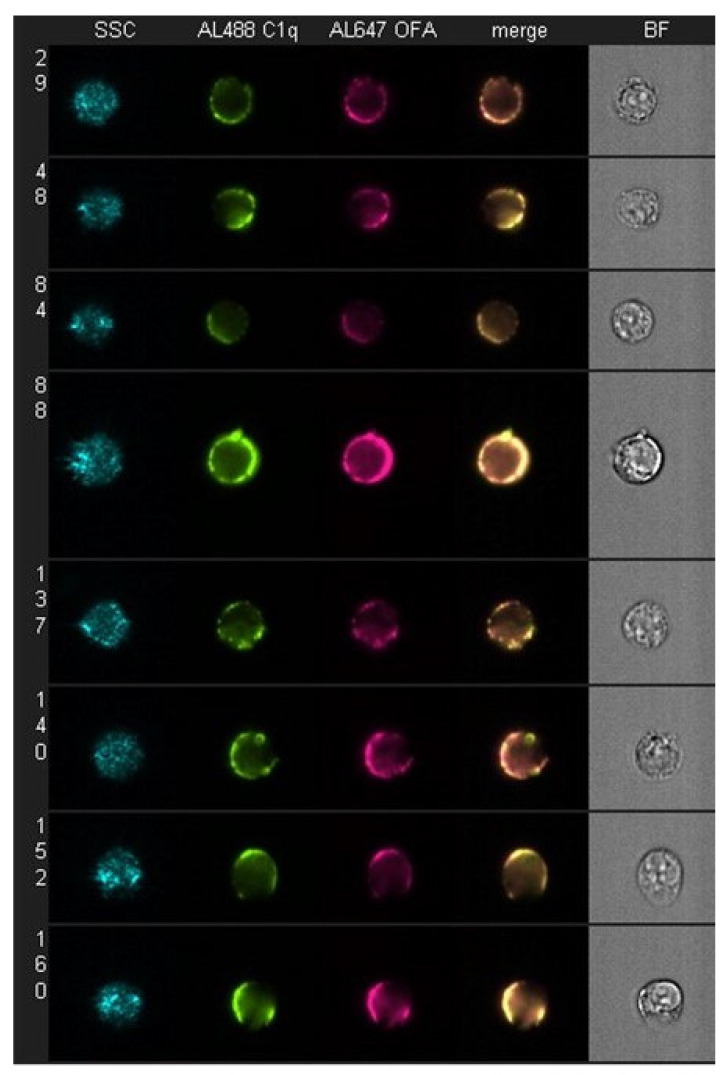
**C1q colocalizes with bound OFA on daudi cells.** Daudi cells were incubated with 10 μg/mL Al647 mAb and 1.6 μg/mL Al488 C1q in medium for 60 min at 37 °C. Samples were washed, fixed, concentrated, and then analyzed by multispectral high-resolution digital imaging (HRDI). Fluorescence signals for Al647 OFA-opsonized samples were analyzed for colocalization with bound Al488 C1q. Light scatter, fluorescence and bright field images of representative doubly positive cells. Results are representative of three similar experiments. Figure 1 and Figure 2 were originally published in *The Journal of Immunology*. Pawluczkowycz, A.W. et al. 2009 Binding of submaximal C1q promotes CDC of B cells opsonized with anti-CD20 mAbs OFA or RTX: considerably higher levels of CDC are induced by OFA than by RTX. *J. Immunol.* 183: 749–758. Copyright © (2009) The American Association of Immunologists, Inc. [17].

It is well established that during C activation, innocent bystander opsonization or lysis of nearby non-targeted cells is negligible [4,39,40,41]. Therefore, implicit in the specificity of the lytic C cascade is the presumption that there is both concentration and localization of activated C components at the nexus of C activation, which, for the classical pathway, would be the cell-bound mAbs at the plasma membrane target site (e.g., CD20). We have demonstrated this phenomenon of colocalization of reactive proteins at multiple steps in the pathway, starting with C1q binding [17]. First, we observed a high level of colocalization of Al647 OFA with Al488 C1q on Daudi cells quantitated by HRDI measurements (Figure 2 and Table 2). However, when we performed the experiment under identical conditions with Al647 RTX-opsonized Daudi cells, we found that although the amount of cell-bound RTX was comparable to the amount of cell-bound OFA, there was a very modest level of C1q binding (Table 2) and considerably less colocalization of cell-bound C1q with RTX. Based on analyses of their CD20 epitope specificities, it is now well established that OFA binds much closer to the cell membrane and with a slower off rate than RTX [19,31,42,43,44], and this has also been reflected “downstream” in that OFA is able to mediate CDC of B cells much more effectively than does RTX. This is particularly apparent in the case of ARH77 cells and CLL B cells. These cells are more resistant to mAb-induced CDC than most B cell lines because they express low levels of CD20 but high levels of C control proteins CD55 and/or CD59, and therefore the differences in the CDC efficacy of OFA versus RTX are readily demonstrated [19,31,43,44].

Table 2 was originally published in *The Journal of Immunology*. Pawluczkowycz, A.W. et al. 2009 Binding of submaximal C1q promotes CDC of B cells opsonized with anti-CD20 mAbs OFA or RTX: considerably higher levels of CDC are induced by OFA than by RTX. *J. Immunol.* 183: 749–758. Copyright © (2009) The American Association of Immunologists, Inc. [17].

**Figure 3:**
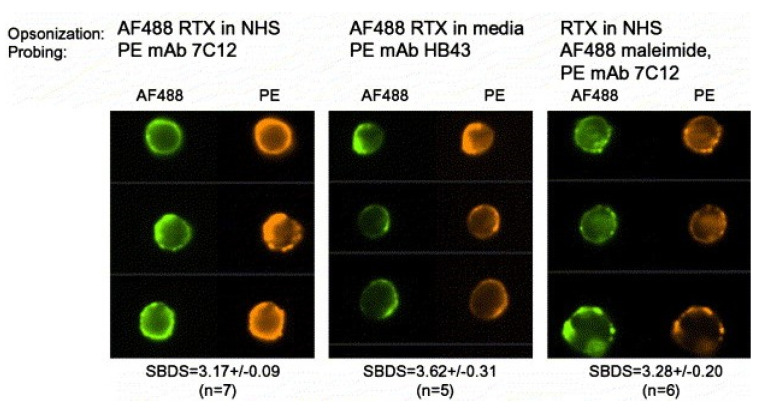
**Deposited C3b colocalizes with bound RTX.** Representative images from samples opsonized as indicated and then analyzed by HRDI. Similarity bright detail score (SBDS) values given below the images are the mean ± SD (*n*) for values obtained on ‘*n*’ different replicate samples (at least 10,000 cells/sample) prepared and analyzed over a period of 18 months. The AF488 maleimide binds to the free SH group on C3b. Note how it is colocalized with mAb 7C12, specific for C3b. Reprinted from Journal of Immunological Methods, Vol. 317, 2006, Beum, P.V. et al., Quantitative analysis of protein colocalization on B cells opsonized with rituximab and complement using the ImageStream multispectral imaging flow cytometer, pp. 90–99, with permission from Elsevier, Amsterdam, The Netherlands [18].

These findings also speak to the issue of thresholds for C activation [9,45,46] and cell killing by the MAC. Binding of RTX to B cells does indeed allow for some C1q binding, C activation, and subsequent C3b deposition and colocalization of the deposited cell-bound C3b with cell-bound RTX (Figure 3) [18,19]. However, we found that on reaction in NHS, the amount of C3b deposited on OFA-reacted CLL cells was 5–10-fold greater than the amount of C3b deposited on RTX-opsonized CLL cells, quantitated with flow cytometry measurements [31]. Thus, although there is comparable binding of these CD20 mAbs to the CLL cells and there is enough C1q bound to RTX-opsonized cells to activate C, less C3b is deposited on the cells compared to the amount of C3b deposition mediated by OFA [17,31]. In other words, the C3b deposition threshold needed to achieve *adequate* generation of the MAC to enable cell killing is not reached for most RTX-opsonized CLL cells. It is therefore understandable why OFA is considerably more effective than RTX in promoting CDC of CLL cells.

However, RTX can promote very rapid C3b deposition on B cells in the circulation of non-human primates. We found that when RTX is infused intravenously into cynomolgus monkeys, it rapidly binds to circulating B cells and this is followed, within 2 min, by deposition of C3 fragments in close juxtaposition with B cell-bound RTX [36]. Moreover, we also obtained blood samples from CLL patients treated with either RTX or OFA, and we observed C3 fragments deposited on their B cells in close juxtaposition with cell-bound RTX/OFA on samples taken within an hour of the start of the mAb infusions [31,47,48]. We note that multiple lines of evidence indicate that mechanisms mediated by receptors for the Fc region of human IgG (FcγR) expressed on macrophages are principally responsible for the in vivo efficacy of RTX [49,50,51].

## 5. C3b Deposition Kinetics, a Key Intermediate Step in CDC; the “Discovery” of Streamers

**Figure 4:**
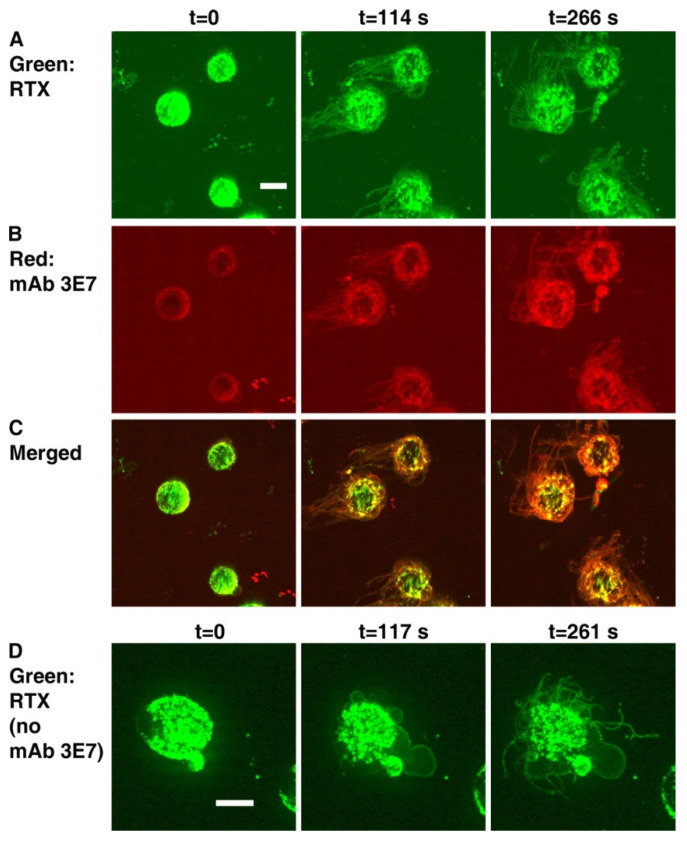
**Binding of RTX to Daudi cells in NHS induces blebbing and streaming.** (**A**–**C**), Images obtained at three different times for Daudi cells reacted with Al488 RTX, Al647 mAb 3E7, and NHS. (**A**), The 488 nm images show green RTX. (**B**), The 647 nm images show red mAb 3E7. (**C**), Merged images. Note that overlap of red and green produces orange or yellow. (**D**), Blebbing and streamers are generated in the absence of mAb 3E7. Daudi cells were opsonized with Al488 RTX and then reacted with NHS. Images for 488 nm are displayed at three time points. Green streamers can be seen to the left of the cells in panel A. The calibration bar in this and the following figures denotes 5 microns. The magnification in (**A**–**C**) was 40×, but, in panel (**D**) and all other figures derived from spinning disc confocal microscopy experiments, magnification at 63× was used [19].

We next asked whether the kinetics of C3b deposition and killing of B cell lines or of CLL cells opsonized with these CD20 mAbs would also reflect differences between OFA and RTX. We conducted real-time spinning disk confocal fluorescence microscopy experiments in which Alexa-labeled mAbs specific for CD20 were reacted with B cells and then incubated in NHS as a C source supplemented with Alexa-labeled mAb 3E7 as a marker to reveal C3b/iC3b deposition. Importantly, mAb 3E7 does not cross-react with C3 and therefore can report C3b deposition in situ. [19]. We confirmed that under these conditions, both RTX and OFA promoted rapid C activation (~2 min), and that substantial colocalization of deposited C3b with cell-bound RTX was easily demonstrable on Daudi cells (Figure 4, panels A–C). However, an unexpected and initially very puzzling observation set the stage for more detailed investigations that have ultimately allowed us to carefully decipher the intricacies of the CDC killing mechanism for nucleated cells. We found that *very soon after* the C3b deposition reaction could be detected, long very thin fragments of cell membrane extended from the Daudi cells *before* they were killed, and it was possible to detect both membrane-bound mAb (RTX or OFA) along with colocalized C3b on these fragments. Control experiments in the absence of mAb 3E7 clearly demonstrated that these structures were not an experimental artifact (Figure 4, panel D). We initially called these membrane fragments “streamers”, but additional experiments revealed that we were studying the formation of tunneling nanotubules (TNTs), in a reaction that is mediated by rapid entry of large amounts of Ca^2+^ into a cell [22,52,53].

**Figure 5:**
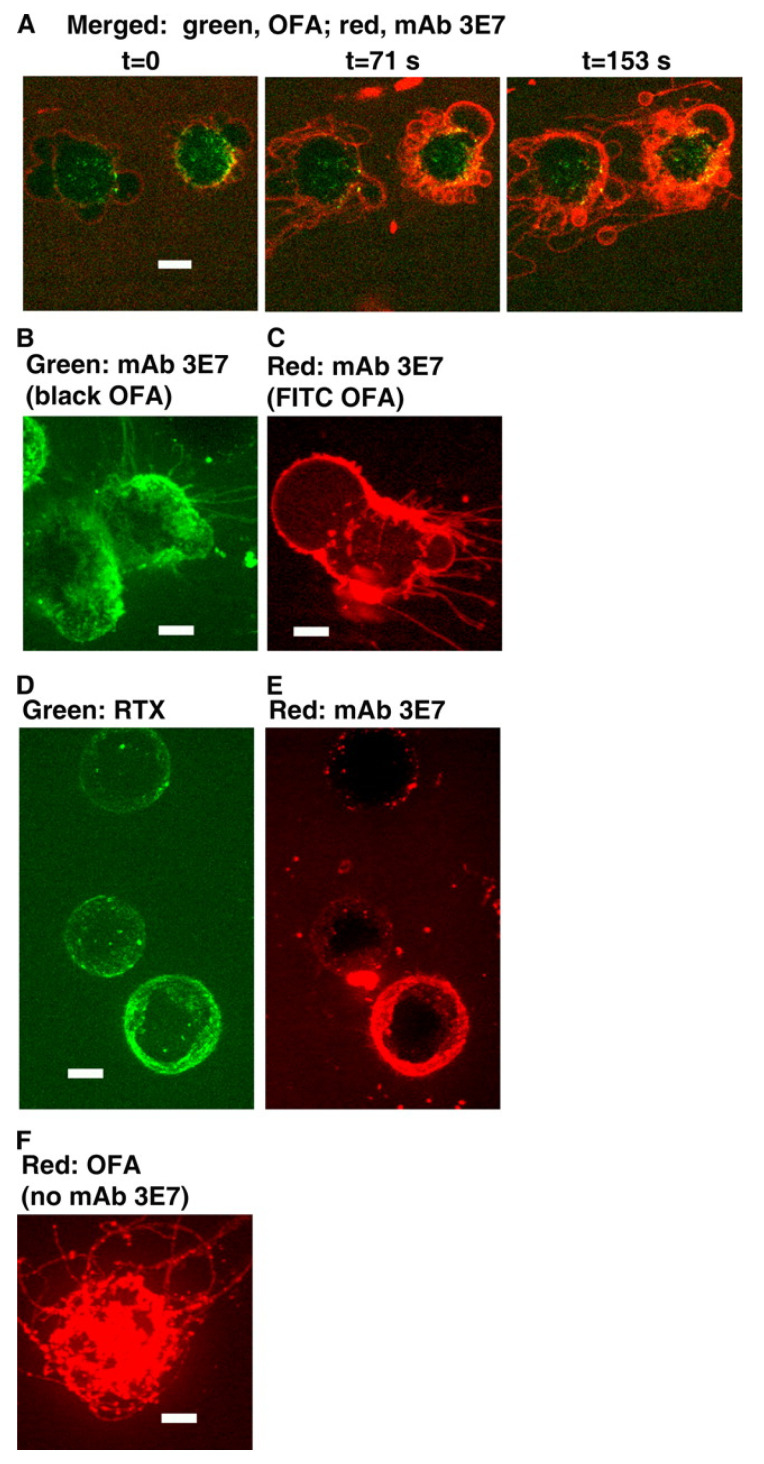
**Binding of OFA to Daudi cells and ARH77 cells in NHS produces blebbing and streamers.** (**A**), Daudi cells were opsonized with Al488 OFA, and then reacted with NHS and Al647 mAb 3E7; merged 488 nm/647 nm images at the indicated times. (**B**), ARH77 cells were opsonized with OFA, and then reacted with NHS and Al488 mAb 3E7; the 488 nm image shows mAb 3E7. (**C**), ARH77 cells were opsonized with FITC OFA, and then reacted with NHS and Al647 mAb 3E7; the 647 nm image shows mAb 3E7. (**D**,**E**), ARH77 cells were opsonized with Al488 RTX, and then reacted with NHS and Al647 mAb 3E7; the 488 nm image (**D**) shows RTX and the 647 nm image (**E**) shows mAb 3E7. There is neither blebbing nor streamers in panels (**D**,**E**). (**F**), Blebbing and streamers are generated in the absence of mAb 3E7. Daudi cells were opsonized with Al647 OFA and then reacted with NHS. The 647 nm image is displayed. Figure 4 and Figure 5 were originally published in *The Journal of Immunology*. Beum, P.V. et al., 2008, Complement activation on B lymphocytes opsonized with rituximab or ofatumumab produces substantial changes in membrane structure preceding cell lysis, *J. Immunol.* 181:822–832. Copyright © (2008) The American Association of Immunologists, Inc. [19].

We performed a similar experiment substituting ARH77 cells for Daudi cells. As noted previously, OFA, but not *RTX*, can mediate CDC of ARH77 cells. We observed colocalization of OFA or of RTX with C3b on ARH77 cells when the cells activated C in the presence of NHS. However, TNTs/streamers were released only by OFA-reacted ARH77 cells, but not by ARH77 cells reacted with RTX (Figure 5, panels A–D) [19]. This finding strongly suggests that C activation on ARH77 cells induced by RTX was not adequate to promote entry of Ca^2+^ into these cells. That is, in view of the large differentials in C3b deposition and cell killing for OFA versus RTX-opsonized CLL cells, these findings again support the idea that the threshold for C3b deposition required for generation downstream of sufficient amounts of the MAC to effectively permeabilize ARH77 cells and promote Ca^2+^ entry is not reached for RTX-reacted ARH77 cells. The patterns we have described (colocalization of mAb and C3b, formation of TNTs) are not unique to CD20 mAbs. Certain mAbs activate C very effectively on binding to target cells because they bind at very high levels (>80,000 mAbs per cell). These include HB28 (anti-β2 microglobulin, mouse (IgG2b), alemtuzumab (anti-CD52), and W6/32 (anti-HLA), and all of these mAbs have also been demonstrated to produce streamers/TNTs on binding to target cells in the presence of C [19,22,33,54].

## 6. On the Importance of Ca^2+^

**Figure 6:**
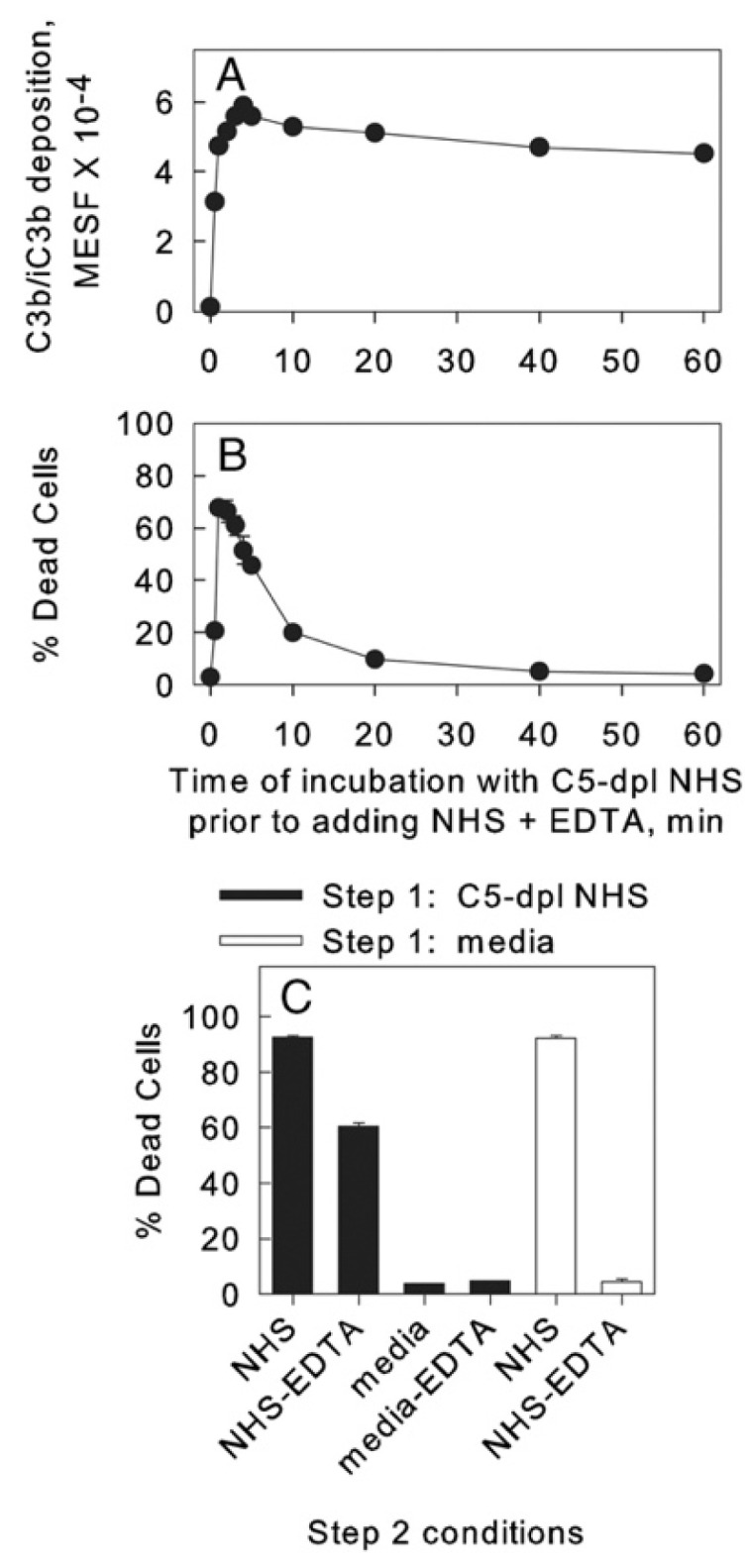
**Two-step CDC.** Reaction of OFA-opsonized Daudi cells in C5-depleted (C5-dpl) NHS in step 1 promotes C3b/iC3b deposition and these cells are killed when they are incubated in either NHS or NHS + EDTA in step 2. (**A**) C3b/iC3b deposition was assayed with PE mAb 7C12 and is expressed as molecules of equivalent soluble fluorochrome (MESF). In a control to block C3b deposition, cells were opsonized in step 1 with C5-depleted NHS + EDTA; the resulting signal was only 1200 MESF. (**B**) The percentage of dead cells was determined based on uptake of TO-PRO-3. (**A**,**B**) Representative of five similar experiments. (**C**) Killing of cells under various step 2 conditions. Means and SD are displayed. Representative of three similar experiments [22].

**Figure 7:**
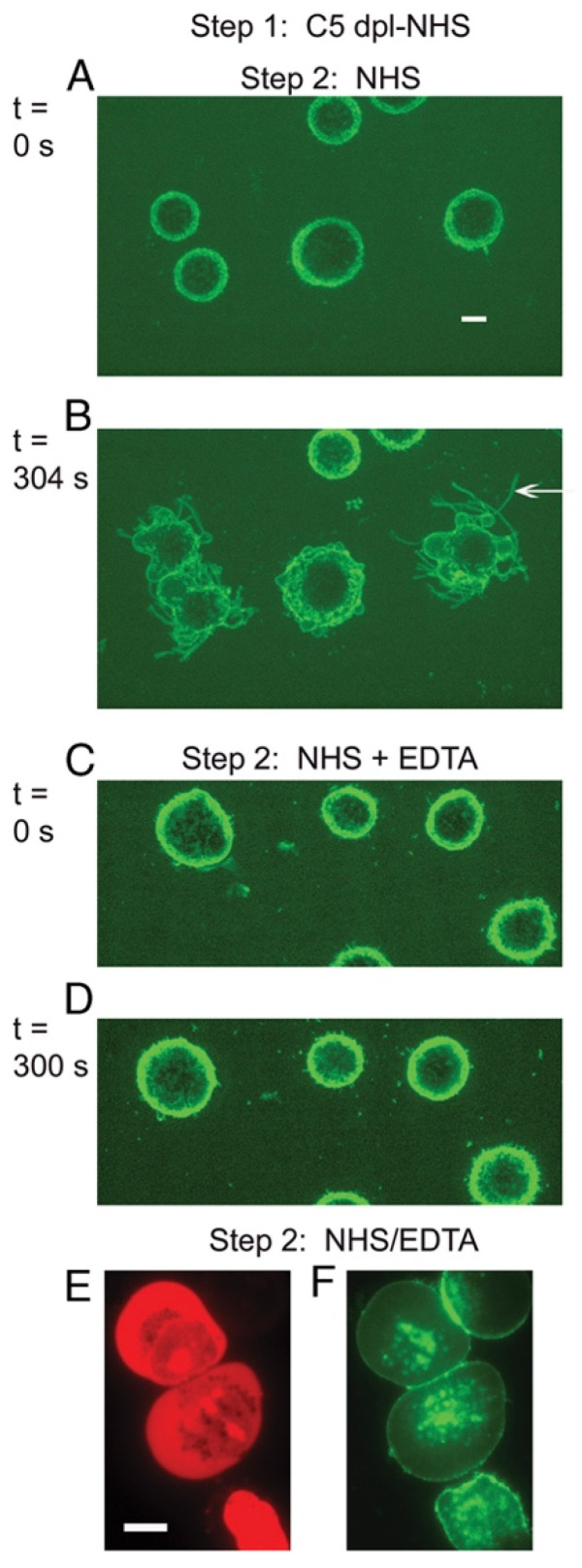
**Production of streamers requires Ca^2+^.** OFA-opsonized cells reacted with C5-depleted NHS do not exhibit streaming when they are incubated with NHS + EDTA. (**A**–**D**) Al488 mAb 3E7 was used to visualize the C3b-opsonized cells, and representative images from spinning disc confocal microscopy (SDCM) movies are displayed at time 0 (**A**,**C**) and after 304 or 300 s (**B**,**D**). A streamer in (**B**) is identified by the arrow. Images shown are representative of more than 20 similar experiments. Magnification at 63× in all images. Scale bar = 5 microns. (**E**,**F**) Fluorescence microscopy (FM) analysis of Al488 OFA-opsonized Daudi cells subjected to the two-step protocol and then stained with PI to identify dead (stained red) cells. Although the cells are dead (**E**) there is no evidence for blebbing or steamers. Magnification at 100×; scale bar = 10 microns. Representative of more than 15 fields examined in two separate experiments. Figure 6 and Figure 7 were originally published in the *European Journal of Immunology*, Vol. 41, Beum, P.V. et al. 2011, Penetration of antibody-opsonized cells by the membrane attack complex of complement promotes Ca^2+^ influx and induces streamers. *Eur. J. Immunol.* pp. 2436–2446, © 2011 Wiley-VCH Verlag GmbH & Co. KGaA, Weinheim [22].

Based on these observations, we next focused on investigating the possible role of Ca^2+^ in the cell-killing phase of the CDC reaction [22]. Upstream deposition of C3b occurs in a process which requires Ca^2+^, but the downstream terminal steps in the C cascade, in particular, generation of the MAC, do *not* directly require Ca^2+^. Therefore, we briefly reacted Daudi cells with OFA in C5-depleted serum to deposit active C3b but not permit subsequent MAC formation. The cells were then washed and incubated in NHS-EDTA (to chelate Ca^2+^) or in NHS. Under both conditions, the MAC is then generated and the cells are killed (Figure 6); however, TNTs are not produced when the cells are killed in NHS-EDTA, providing strong evidence that in NHS it is entry of Ca^2+^ into the cells that promotes TNT formation (Figure 7) [22]. The degree of killing for C3b-opsonized cells reacted in NHS-EDTA was somewhat lower and slower than in in NHS. We suggest that under these conditions, where Ca^2+^ entry into cells is precluded, we are instead studying “death by drowning” of the cells due to influx of large amounts of water and loss of cellular constituents upon permeabilization of the cell membrane. However, it is our working hypothesis that when the cells are killed under normal physiological conditions for CDC, it is the influx of lethal amounts of Ca^2+^ that provides the most immediate fatal blow. A similar finding of slower cell killing was reported by Papadimitriou et al., who examined MAC killing of EACs in the presence of EGTA in which Ca^2+^ was chelated, thus precluding its rapid entry into the cells [14,15].

At this point, our research direction was strongly influenced by a general theory as to how toxins kill cells, developed more than 40 years ago by Schanne et al. [55]. They noted that in a first step, toxin could compromise the integrity of cell membranes by a variety of mechanisms that were usually independent of Ca^2+^. However, they suggested that “the second step of toxin induced killing most likely represents an influx of Ca^2+^ across the damaged plasma membrane … and represents a final common pathway by which the cells are killed.” Indeed, under normal physiological conditions, the external extracellular Ca^2+^ concentration in blood and interstitial fluid is in the millimolar range, but the Ca^2+^ concentration in most nucleated cells is approximately 0.1 uM, and levels above 2 uM are usually lethal [9,56,57,58]. Therefore, it is quite reasonable to expect that when the plasma membrane of a cell is effectively permeabilized by the MAC “toxin”, the cell will then be killed as a consequence of influx of Ca^2+^ and poisoning of many of its major metabolic pathways due to Ca^2+^-mediated excessive activation of a variety of proteases, endonucleases, and phospholipases [12,14,15].

## 7. Hexamer-Forming mAbs are More Effective in Activating C

### 7.1. Cell-Bound Hexamer-Forming mAbs Bind C1q

In a very productive collaboration, we made use of hexamer-forming mAbs that were developed by Prof. Paul Parren and his colleagues at Genmab [16,20,59,60,61,62]. We used these mAbs to further study the role of Ca^2+^ entry into cells in the mAb-mediated CDC reaction. These mAbs are modified in the Fc region, which substantially enhances their potential to form hexamers upon binding to cells. The modifications (e.g., E430G) promote much more effective and rapid binding of the classical pathway initiating factor, hexameric C1q, to the mAb-opsonized cells, and thereby increase the CDC potential of a wide range of mAbs. We also note that numerous other strategies are under investigation for increasing the ability of mAbs to promote CDC of tumor cells [26,27,41,63,64,65,66,67,68,69,70,71,72]. It will be interesting to compare the efficacy of these strategies if they progress to clinical trials.

**Figure 8:**
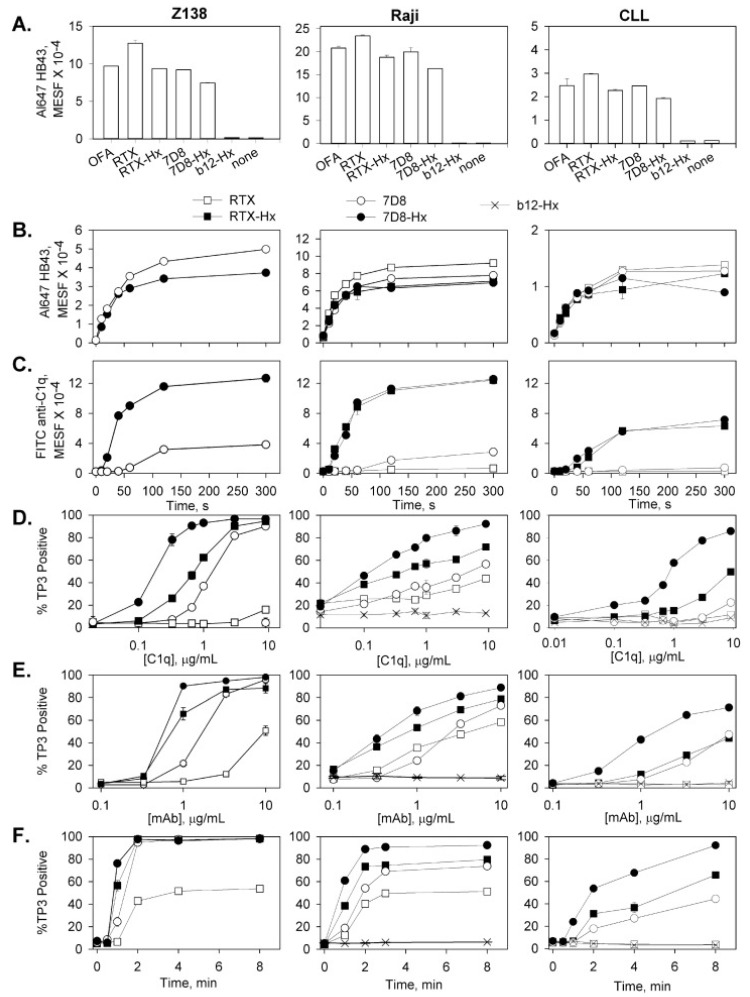
**Hexamer-forming CD20 mAbs efficiently utilize small amounts of C1q to mediate CDC.** Hexamer-forming CD20 mAbs bind to B cells and promote C1q binding, CDC and efficiently utilize small amounts of C1q to mediate CDC. (**A**) Saturating amounts of mAbs were reacted with cells for 30 min at RT in media, and after two washes, binding was evaluated by development with Al647 mAb HB43 (10 μg/mL, 30 min on ice), specific for human IgG Fc region. All points are in duplicate, and means and SD are displayed. (**B**,**C**) Kinetics of mAb binding and C1q uptake were determined based on reacting mAbs (10 μg/mL) with cells in 5% NHS for varying times at 37 °C. After washing, development was based on probing with (**B**) Al647 mAb HB43 or (**C**) FITC anti-C1q antibody. (**D**) CDC with 10 μg/mL mAb in 50% C1q-depleted serum supplemented with varying amounts of C1q for 20 min at 37 °C. % TO-PRO-3 (TP3)-positive cells define % CDC. (**E**) Cells were reacted with mAbs at 37 °C for 20 min in 50% NHS, and CDC was evaluated after TO-PRO-3 staining. (**F**) Kinetics of killing were determined after reacting cells with 10 μg/mL mAb in 50% NHS for varying times. (**A**–**F**) Each graph is representative of 2–4 similar experiments. Results for CLL cells shown in this figure were obtained with cells from four CLL patients. Cytotoxicity for CLL cells reacted in the absence of mAbs, or plus mAb in media, or in heat-inactivated NHS, was less than 6% [16].

We first compared 7D8 (a close analogue of OFA [73]), RTX and the hexamer-forming variants, 7D8-Hx and RTX-Hx. Although approximately comparable amounts of the different CD20 mAbs bind to a given cell, 7D8-Hx and RTX-Hx are considerably more effective at promoting C1q binding and rapid C-mediated killing of the target cells (Figure 8) [16]. We also found that all four mAbs bound to the cells within 1–2 min.

### 7.2. Four-Color Confocal Microscopy Movies

**Figure 9:**
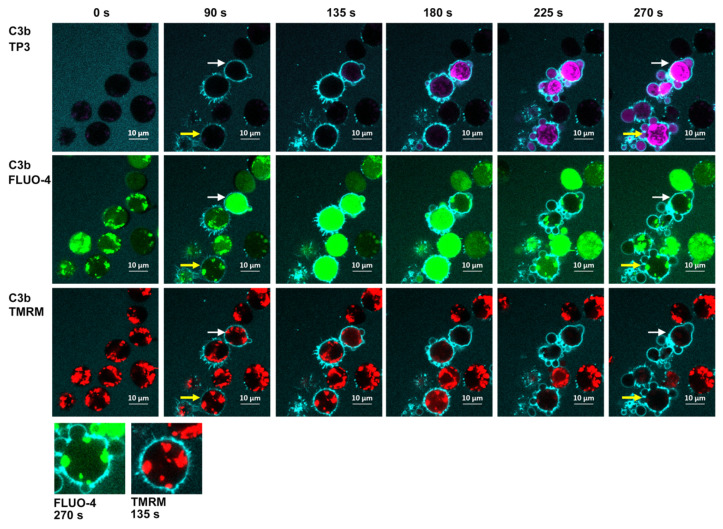
**Four-color confocal fluorescence microscopy analyses of the kinetics of CDC of Raji cells mediated by 7D8-Hx.** Delineation of discrete steps in CDC of Raji cells: four-color confocal fluorescence microscopy analyses of the kinetics of CDC of Raji cells mediated by 7D8-Hx. For clarity, images based on analyses with two colors at advancing times during the reaction are displayed. Upper panel: Al405 mAb 3E7 (C3b/iC3b, light blue) and TO-PRO-3 (dead cells, bright purple). Middle panel: Al405 mAb 3E7 (C3b/iC3b) and Fluo-4 (Ca^2+^, green). Lower panel: Al405 mAb 3E7 (C3b/iC3b) and TMRM (viable mitochondria red). White arrows mark a representative cell in the transition state at 90 s and dead at 270 s. Yellow arrows identify a cell in which the mitochondria remain viable through the transition-state intermediate. Inset: Amplified image of a single cell at 270 s (Fluo-4) and 135 s (TMRM). Note that the green signal due to Fluo-4 in the dead cell (at 270 s) is localized to places in the live cell in which viable mitochondria (positive TMRM staining) are identified at 135 s. Representative of more than 10 similar experiments [16].

We next performed a series of kinetic experiments to study CDC-mediated by 7D8-Hx based on four-color confocal microscopy movies. Raji B cells or CLL cells were internally labeled with the green fluorescent Ca^2+^ indicator, Fluo-4, to monitor Ca^2+^ influx. Viable mitochondria were visualized with TMRME (red). The cells were dispersed in NHS containing Alexa-405-labeled anti-C3b/iC3b mAb 3E7 (blue) to follow C3b deposition and TO-PRO-3 (purple) was added to the medium as a live/dead indicator. The screenshots (Figure 9) [16] from the movies of Raji cells reacted with 7D8-Hx demonstrate that many of the cells are opsonized with C3b (binding of blue anti-C3b mAb 3E7) after approximately 90 s. Soon thereafter, enough MAC must have penetrated the cells, because many of them are bright green (indicating influx of Ca^2+^), but some are still alive, and their mitochondria appear to be alive and intact, based on the viable red TMRME signal (135 and 180 s). We have identified these bright green cells as “transition-state intermediates”. They are alive, but are doomed, because lethal amounts of Ca^2+^ had entered the cells. Soon, by 180–270 s, many of the cells are dying or are dead, and this is evident based on three separate criteria: first, most of the Fluo-4 has leaked out of the cells; second, the mitochondria have been poisoned and are no longer stained by TMRME; and third, TO-PRO-3 has entered the cells. Careful inspection of the movies suggested that there was a slight lag (5–10 s) between the coincident leakage of Fluo-4/loss of the TMRME signal, and the final entry of TOPRO-3 into the dead cells. More details on these phenomena can be found in the Figure 9 caption.

### 7.3. Kinetics of CDC Monitored by Multicolor HRDI

**Figure 10:**
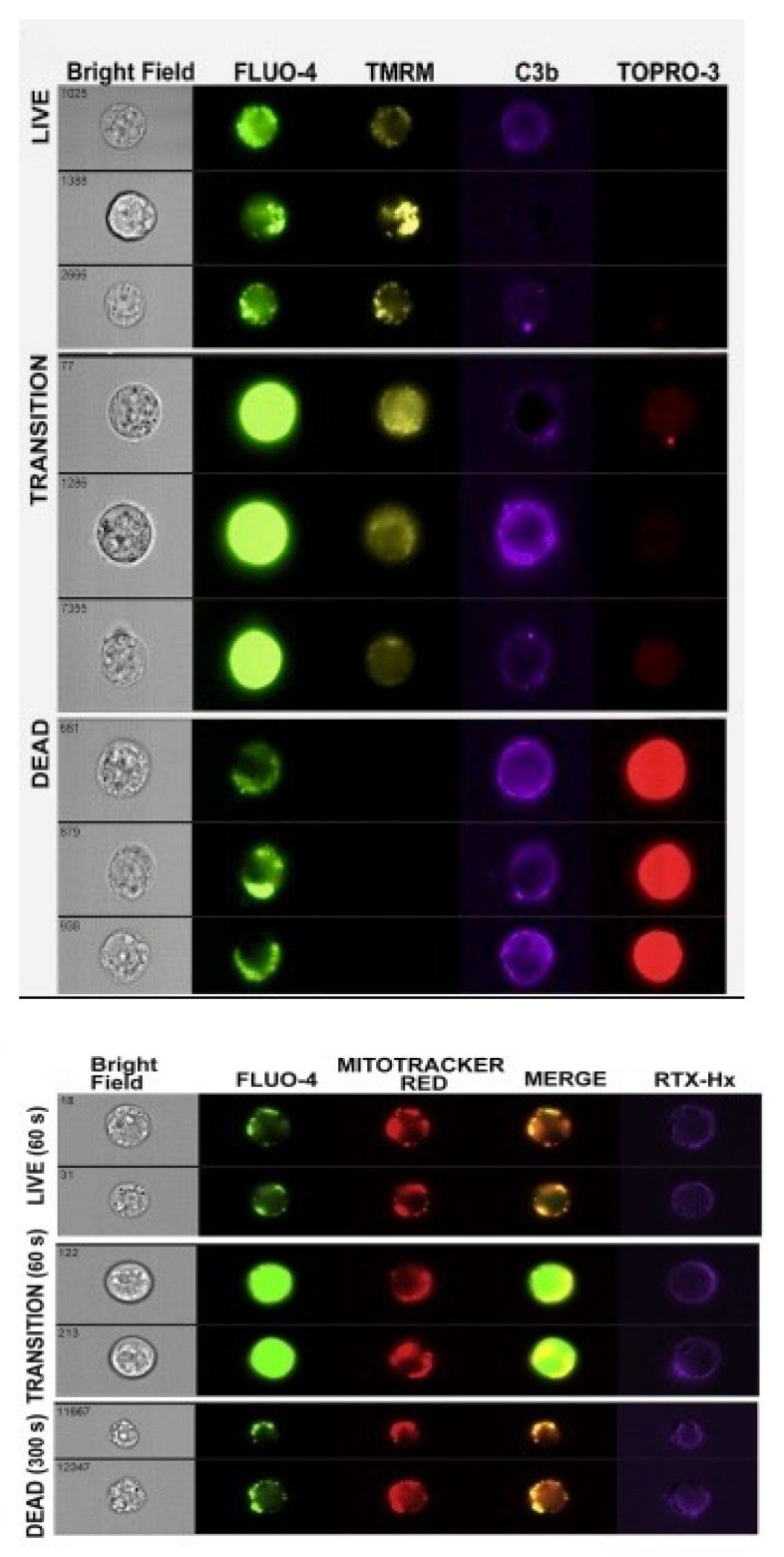
**Four-color kinetic analyses by HRDI of the transition-state intermediate.** Delineation of discrete steps in CDC: Four-color kinetic analyses by multispectral fluorescence imaging. Top panel: Images of representative 7D8-Hx treated cells that are either alive, in transition, or are dead. Bottom panel: Representative images for cells reacted with Al647 RTX-Hx (E345R) after 60 s (live and transition) or after 300 s (dead). The cells were presumed to be dead by 300 s based on the decrease in the mean Fluo-4 signal. Figure 8, Figure 9 and Figure 10 are reprinted from Molecular Immunology, Vol. 70, 2016, Lindorfer, M. A. et al. Real-time analysis of the detailed sequence of cellular events in mAb-mediated complement-dependent cytotoxicity of B cell lines and of CLL B cells, pp. 13–23 with permission from Elsevier [16].

We also made use of HRDI technology to visualize cells during the CDC process. Figure 10, top panel identifies three distinct populations of cells at the 40 s mark that are either: alive—Fluo-4 very bright (transition-state intermediate) and TMRME positive; and finally dead—Fluo-4 weak, TMRME negative, C3b positive and TOPRO-3 positive [16]. Verification that the *residual* Fluo-4 stain is in the mitochondria (identified with Mitotracker Red) of the cells is demonstrated in Figure 10, bottom panel. It is also noteworthy that there is no noticeable swelling of the dead cells (Figure 10, top panel) at early times, soon after they are killed. This again emphasizes that there is no evidence for an early osmotic burst reaction when the nucleated cells are first killed, in agreement with Papadimitriou et al. [15].

**Figure 11:**
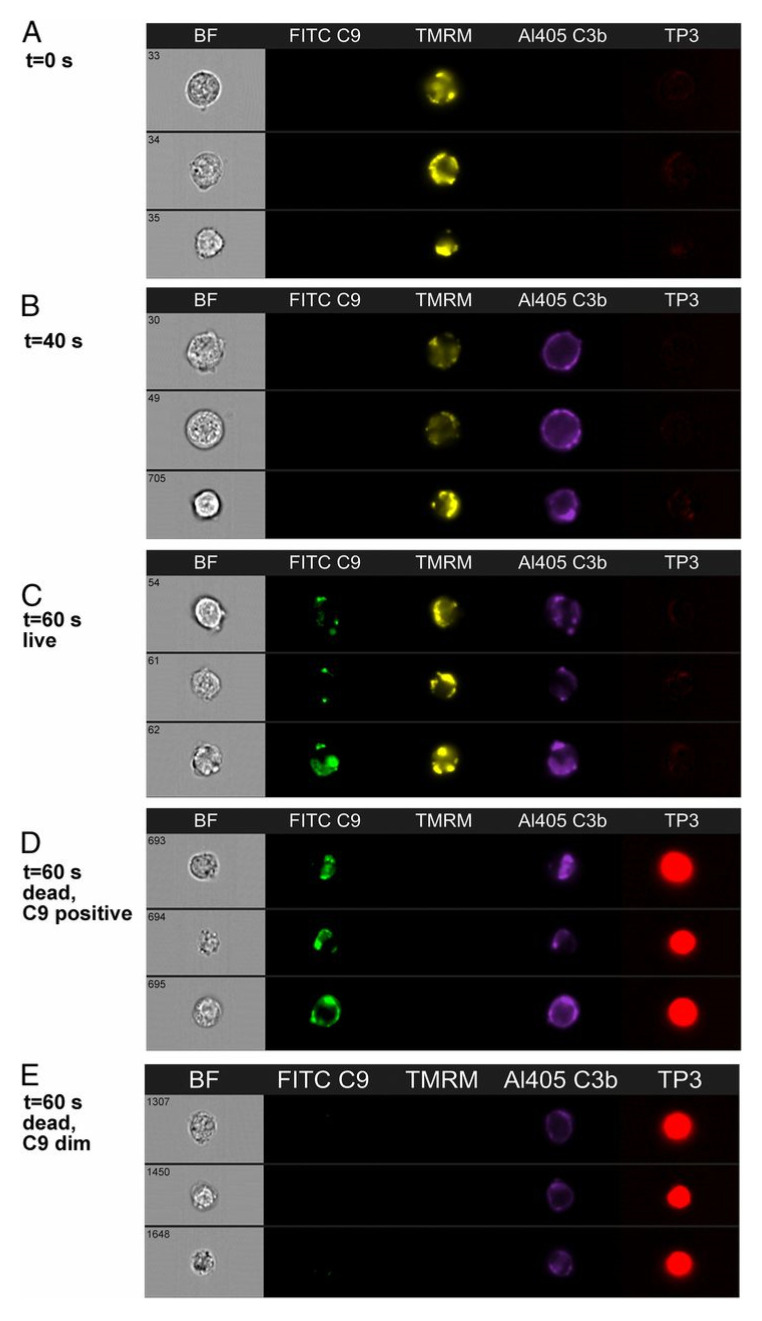
**mAb 7D8-Hx mediates CDC of Z138 cells in C9-depleted NHS.** Distinct cellular steps in the CDC pathway are illustrated with representative images from an experiment in which Z138 cells were reacted with 7D8-Hx in NHS. (**A**) At zero time, cells are alive (TMRM positive, TP3 negative). (**B**) After 40 s, C3b has deposited, but the cells are still alive, based on the positive TMRM signal and the lack of staining by TP3. (**C**,**D**) After 60 s, C9 has bound to the cells. Note that both live (**C**) and dead (**D**) (TP3 positive, TMRM negative) C9-positive cells can be seen. (**E**) After 60 s, 8.6% of dead cells are C9 dim [20].

**Figure 12:**
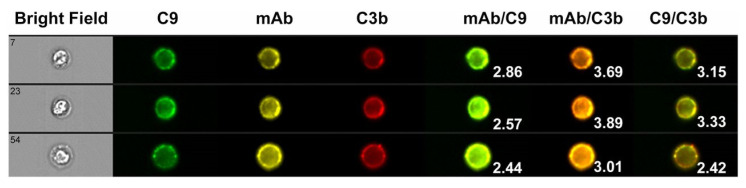
**C3b and C9 are colocalized on B cells opsonized with mAb 7D8-Hx.** C3b and C9 are colocalized on B cells with opsonizing mAb 7D8-Hx after reaction for brief periods in 50% NHS. Data were obtained based on multispectral high-resolution fluorescence imaging by flow cytometry. CLL cells were reacted with Al546 Hx-7D8 in NHS for 8 min and after two washes were probed with both FITC mAb aE11 and with Al594 3E7 (for C3b/iC3b). Images representative of triple-positive cells are shown. Bright detail similarity score (BDSS) values for the merged images of the individual cells are given. A very high degree of colocalization of Hx-7D8 with C3b is evident, and colocalization of the mAb with C9 is also observed [20].

We also used HRDI to follow the CDC reaction for Z138 cells. In these experiments, we monitored C9 binding instead of Ca^2+^ influx. We were able to verify that binding of C9 to the Z138 cells followed C3b deposition, and that live cells containing bound C9 could be identified (Figure 11), but we know that these are also “doomed” cells that will soon experience substantial fluxes of Ca^2+^ [20]. In agreement with earlier studies, there is considerable colocalization of cell-bound mAb with deposited C3b, and in addition deposited C3b clearly serves as a “landing site” for binding of C5b-9, based on the colocalization of C3b and C9 (Figure 12). The identification of the transition-state intermediate (very bright homogeneous Fluo-4 signal) raised an interesting question: could we better validate its existence and stabilize it by slowing leakage of Fluo-4 out of the cell? The coincident question raised in these studies centered on the role of C9; is C9 essential to kill the cells as part of the MAC?

## 8. On the Role of C9

**Figure 13:**
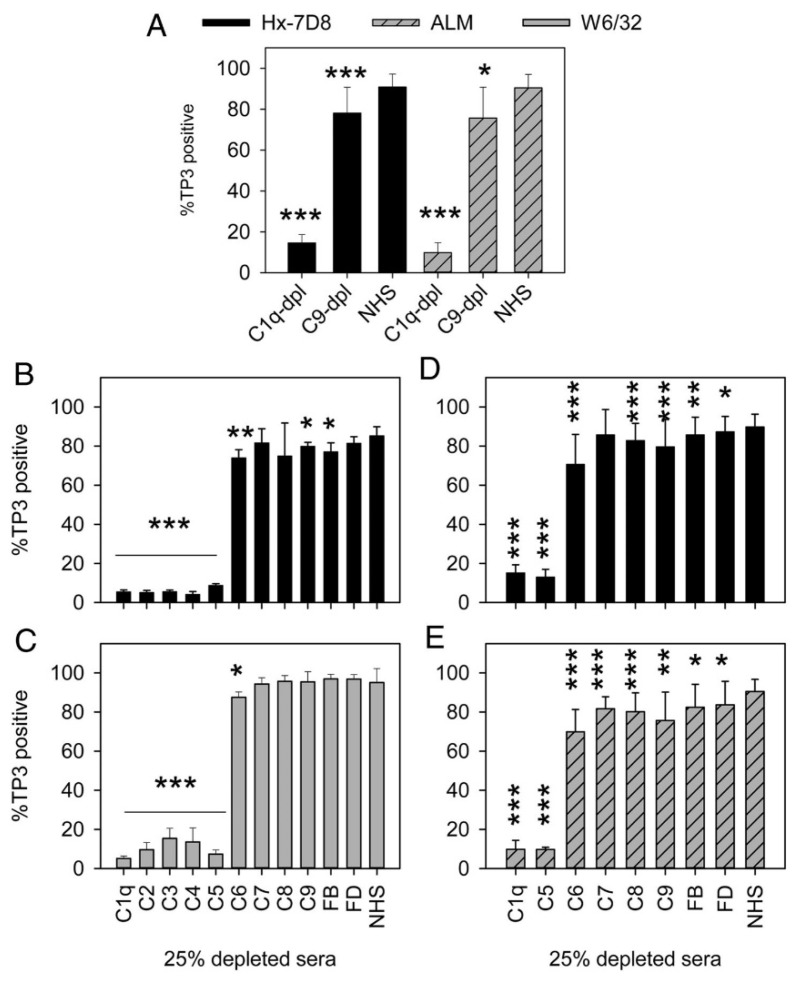
**C9-depleted sera support CDC mediated by several different mAbs.** Sera depleted of one of several terminal (but not upstream) classical pathway complement components support CDC mediated by several different mAbs. (**A**) mAbs Hx-7D8 and ALM promote robust CDC of CLL cells after reaction for 15 min in 25% NHS or C9-dpl sera. The results are the average for duplicate determinations on cells from eight different CLL patients for Hx-7D8 and the average for cells from four of the eight patients for ALM; means and SD are displayed. No CDC is observed if Hx-7D8 or ALM is reacted with CLL cells in C1q-dpl sera. Differences between C1q-dpl and C9-dpl versus NHS are significant, as illustrated. (**B**,**C**) Both Hx-7D8 and W6/32 mediate CDC of Z138 cells in sera depleted of terminal pathway components or of complement factor B (FB) or complement factor D (FD). *n* = 4–6. Significant differences versus NHS are noted. (**D**,**E**) Both Hx-7D8 and ALM mediate CDC of CLL cells in sera depleted of terminal pathway components or of FB or FD. The averaged results for duplicate determinations on cells from eight different CLL patients are provided. The results for C1q-dpl, C9-dpl, and NHS are the same as in (**A**), and are repeated to allow for ready inspection and comparison with the other depleted sera. * *p* < 0.05, ** *p* < 0.01, *** *p* < 0.001. Figure 11, Figure 12 and Figure 13 were originally published in *The Journal of Immunology*. Cook, E. M. et al. 2016, Antibodies that efficiently form hexamers upon antigen binding can induce complement-dependent cytotoxicity under complement-limiting conditions. *J. Immunol.* 197:1762–1775. Copyright © (2016) The American Association of Immunologists, Inc. [20].

We identified small populations of cells that were killed by CDC but did not appear to be stained by C9 [20] (Figure 11, panel E), and we also found that CLL cells and Z138 cells reacted with 7D8-Hx could be killed by CDC in C9-depleted serum. In fact, we reported that the cells could also be killed in sera depleted of Factor B and Factor D (Figure 13). Other mAbs that promote high levels of CDC, including alemtuzumab and W6/32 (Mouse IgG2b, anti-HLA) also were able to mediate CDC in C9-depleted serum, but in all cases mAb-mediated CDC had an absolute requirement for C1q. These findings suggest that the alternative pathway of C (APC) does not appear to be needed to promote effective CDC mediated by the mAbs under investigation. It is generally believed that the APC, based on its inherent exponential amplification loop is responsible for most of the efficacy of C [74,75]. Our findings would suggest this might not be the case for CDC of tumor cells mediated by mAbs, where it appears that the classical pathway (C1q requirement) is key.

**Figure 14:**
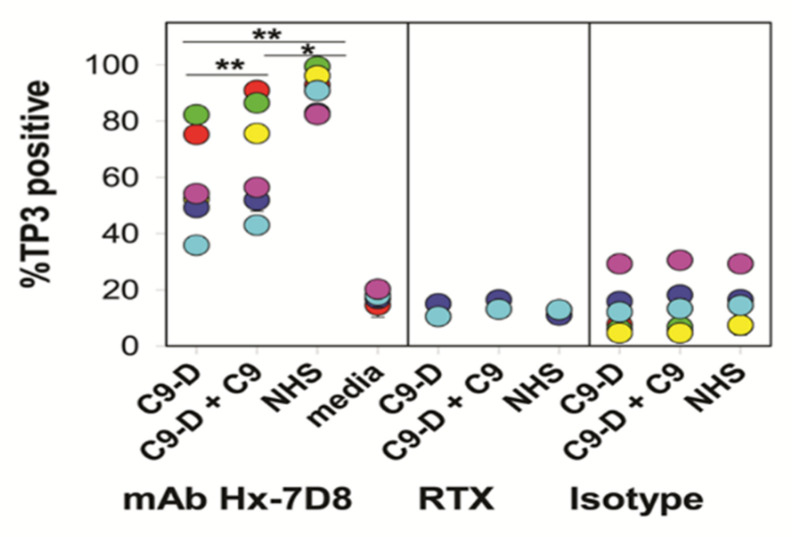
**C9-deficient serum can mediate high levels of CDC of CLL cells opsonized with mAb Hx-7D8.** C9-deficient (C9-D) serum lacks any detectable C9, but can mediate high levels of lysis of CLL cells reacted with mAb Hx-7D8. The ability of mAb Hx-7D8 to promote CDC in CLL cells from six different patients was evaluated in either: C9-D serum (25%), C9-D serum (25%) supplemented with 18 μg/mL C9, or NHS (25%). Controls included Hx-7D8 reacted with cells in media (4 of 6 CLL cell samples) as well as cells reacted with RTX (2 CLL samples) or an isotype control (Isotype, Hx-b12, 2–4 CLL samples) reacted with CLL cells in all three conditions. Each color represents a different patient (pn); all determinations were in duplicate or triplicate and means and SD are provided. Mean % CDC and SD: 58 ± 17% for CLL cells reacted in C9-D serum; 67 ± 20% for CLL cells reacted in C9-D serum + C9; 91 ± 7% for CLL cells reacted in NHS. * *p* < 0.05; ** *p* < 0.01 [21].

**Figure 15:**
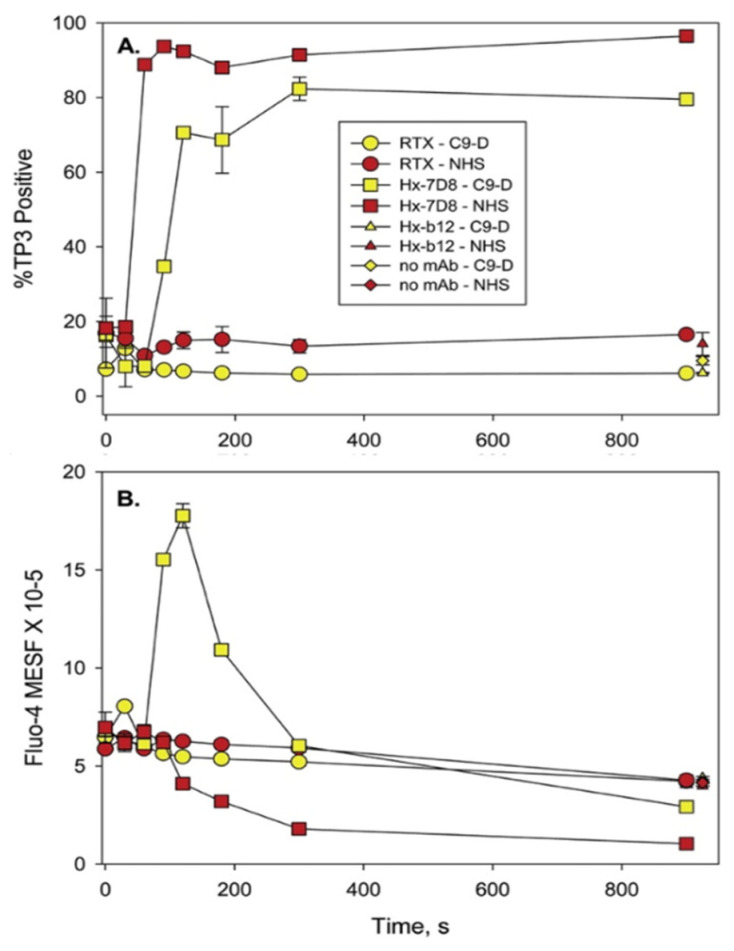
**Analysis of CDC and Ca^2+^ fluxes in Fluo-4-loaded CLL cells.** (**A**) CDC kinetics mediated by Hx-7D8 in C9-D serum (pn 2014, red circles, Figure 14) were slower and killing peaked at approximately 80%, compared to results in intact NHS where killing reached 95%. CDC mediated by RTX was low under both conditions. (**B**) During CDC mediated by Hx-7D8 (also pn 2014), the Fluo-4 signal is increased considerably and remains substantially elevated for 3 min (indicative of the transition-state intermediate) for CLL cells reacted with Hx-7D8 in C9-D serum. Controls include cells reacted for 900 s in NHS or in C9-D serum, with either the isotype control Hx-b12, or with no mAb. Figure 14 and Figure 15 are reprinted from Clinical Immunology, Vol. 181, 2017, Taylor, R.P. et al. Hexamerization-enhanced CD20 antibody mediates complement-dependent cytotoxicity in serum genetically deficient in C9, pp. 24–28, with permission from Elsevier [21].

The results of the experiments with C9-depleted serum are intriguing, but not definitive because trace amounts of C9 could still be present in the depleted serum. However, the smaller C5b-8 pores that penetrate cells are approximately 3.5 nm in size (pores formed with C5b-9 are 10–11.5 nm) and in principle the C5b-8 pores should be adequate to allow for Ca^2+^ entry and killing of the cell [76]. Therefore, in collaboration with Drs. Paul Morgan and Masashi Mizuno, we used flow cytometry to investigate whether CLL cells could be killed by Ca^2+^ fluxes in serum genetically deficient in *C9* [21]. Although we had limited amounts of the C9-deficient serum, we were able to demonstrate that mAb 7D8-Hx promoted substantial CDC of the CLL cells from six different patients (Figure 14). Compared to the reaction in NHS, the CDC kinetics were only slightly slower in the C9-deficient serum. We also asked whether the smaller C5b-8 pores would stabilize the transition-state intermediate (bright green, Fluo-4 very positive) by slowing down exit of Fluo-4 from the cell. Indeed, at the 150 s point, approximately 70% of the cells had been killed, but the net Fluo-4 signal was maximized at this point, indicating that even though most of the cells had been killed due to Ca^2+^ poisoning, the Fluo-4 had not yet leaked out of the cells (Figure 15). The bright green “dead intermediate” was therefore stabilized over a period of approximately 200 s (t = 100 s to 300 s). Due to very limited amounts of C9-deficient serum, we were not able to investigate the reaction with confocal microscopy movies. However, we suggest that further studies with serum from donors genetically deficient in C9 [77] would allow for more comprehensive investigation of these phenomena in the future. For example, it could be quite informative and useful to identify and differentiate tumor cells that are/are not susceptible to CDC in sera lacking C9.

## 9. Ca^2+^ Appears to be the Key: Implications

Our findings, as well as those of the Shin group [10,11,12,13,14,15], provide compelling evidence that it is the influx of Ca^2+^ into nucleated cells that is the primary cause of cell death in the CDC reaction mediated by anti-tumor mAbs. On this basis, there are several additional strategies that could be employed to bring even more efficacy to this process, based on increasing the flux of lethal amounts of Ca^2+^ into the targeted cell. One possibility is also to stimulate the target cell with other mAbs and/or ligands that promote uptake of Ca^2+^ into the cell. For example, Fifadara reported that mast cells costimulated at the high-affinity receptor for the Fc region of IgE (FcεRI) and chemokine receptor 1 (both of which promote Ca^2+^ fluxes) were not killed, but produced TNTs [78]. Apparently, these cells were “on the edge” of being over-stimulated and killed by Ca^2+^, but they survived. If these mast cells were reacted with a mAb that only modestly activated C and the Ca^2+^ flux was inadequate, then perhaps synergy in killing could be achieved by coincident treatment with agents that stimulate FcεRI and/or chemokine receptor 1. This proof-of-concept experiment could be extended to target other stimulatory sites on tumor cells that are known to mediate Ca^2+^ entry. Another approach would be to make use of a mAb–drug conjugate. If a Ca^2+^ ionophore (A23187 or ionomycin) [23] could be stably coupled to a C-fixing mAb with no damage to the potential of the mAb to mediate C activation, then the ionophore could independently further increase Ca^2+^ entry into the targeted cell to increase its cell-killing potential. A similar strategy could be based on coupling pore-forming agents such as melittin or perforin to C-fixing anti-tumor mAbs [23]. Indeed, immunoconjugates of melittin have been investigated for cancer immunotherapy [79]. Finally, as we suggested recently, a third strategy would be to develop a C-fixing antibody drug conjugate that blocks the machinery that pumps Ca^2+^ out of cells, thus synergizing with the CDC action of the anti-tumor mAbs [16].

## 10. The Future

As new and effective C-fixing mAbs are developed for cancer immunotherapy, it is very likely that they will closely follow the patterns we have described here (Table 1). Indeed, in collaboration with investigators at Genmab and Drs. Clive Zent and Richard Burack at the University of Rochester, we recently reported on the properties of CD37 mAbs that contain the E430G hexamer-forming modification [32]. We found that these mAbs promote very high levels (>98%) of CDC of CLL cells taken from newly diagnosed patients. The very high levels of killing are likely due to the fact that most CLL cells express approximately twice as many CD37 epitopes as CD20 epitopes [32,80]. Moreover, based on four-color confocal microscopy real-time movies, we found that upon binding of the CD37-Hx mAb to CLL cells in the presence of NHS, C3b is rapidly colocalized with cell-bound CD37-Hx mAb, and the same pattern of rapid cell killing mediated by Ca^2+^ influx is evident [80]. This includes generation of transition-state intermediates followed by poisoning of mitochondria, leakage of Fluo-4 out of the cell, and finally cell death, all within just a few minutes. We suggest that this cytotoxicity pattern can serve as a “litmus test” for evaluation of the potential of future mAbs intended to be used for cancer immunotherapy based on C-mediated killing.

## 11. Summary

**Figure 16:**
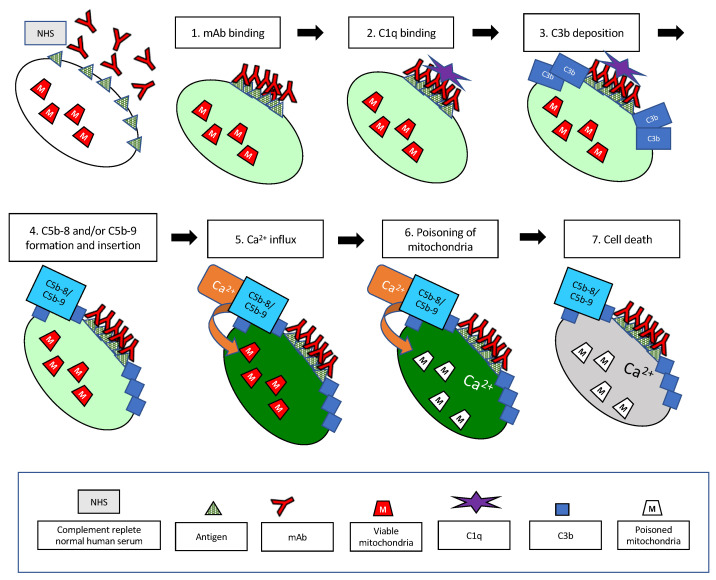
Simplified schematic illustrating the key steps in the CDC reaction that are described in this review. The reaction takes place at 37 °C in 50% NHS, and the mAb is at a concentration of 10 μg/mL. The target cell is internally labeled with Fluo-4. The pale green color of the cell illustrates the low, physiological levels of cytoplasmic Ca^+2^. (**1**). Multiple mAbs bind to the cell in proximity, ideally forming hexamers. (**2**). C1q then binds to the mAb hexamers on the cell. (**3**). Downstream, C3b is deposited on the cell, closely associated with bound mAb. (**4**). After sufficient C3b has deposited on the cell, C5b is generated, and C5b-8 and/or C5b-9 assemble close to the sites of C3b deposition. (**5**). The pores formed in the plasma membrane by C5b-8 and/or C5b-9 allow the influx of large amounts of Ca^2+^ and the cell turns bright green (the live, transition-state intermediate). (**6**). Soon thereafter, the Ca^2^ in the cell penetrates and poisons the mitochondria. (**7**). This is rapidly followed by cell death. Coincident with cell death, most of the Fluo-4 leaks out of the cell. Not shown are the TNTs that are generated in the cells soon after the initial influx of Ca^2+^, during steps (**5**) and (**6**). Approximate times for these events are provided in Table 1, and all of the reactions are fast. The hexamer-forming mAbs and C1q are bound to the cell in less than 1 min, and the cells are killed in less than 2 min.

As illustrated in Figure 16, we have examined and characterized, from the “point of view” of the cell, many of the key steps in the classical complement pathway that are activated when highly effective C-activating mAbs bind to tumor cells. The entire reaction is quite rapid, and both cell lines and primary CLL cells can be killed within just a few minutes due to influx of lethal amounts of Ca^2+^, clearly not allowing much time for any complex signaling pathways to be activated. The patterns we have described are likely to be quite general, and it may be possible to make use of the lessons we have learned in the development of additional innovative strategies that employ C and mAbs in the treatment of cancer and other diseases as well.

## 12. Patents

R.P.T. and M.A.L. are listed as co-inventors on a patent that describes the use of hexamer-forming antibodies.

## Figures and Tables

**Table 1:** Observed consecutive steps in monoclonal antibody (mAb)-mediated complement-dependent cytotoxicity (CDC).

Step	Time Frame *	Method	Figure	References
mAb binding	30 s	Flow cytometry	8	[16]
C1q binding and colocalization with mAb	60 s	Flow cytometry	2, 8	[16,17]
C3b deposition and mAb colocalization	90 s	Flow cytometry, high-resolution digital imaging (HRDI)	3, 4, 10	[16,18,19]
C9 binding and colocalization of C9/C3b/mAb	60–120 s	HRDI, confocal	11, 12	[20]
Ca^2+^ influx, transition-state intermediate	40–135 s	Confocal, flow cytometry	9, 10, 15, 16	[16,21]
tunneling nanotubule (TNT) generation	120 s	Spinning disk	4, 7	[19,22]
mitochondrial poisoning	180 s 60–180 s	HRDI, confocal	9–11	[16,20]
decay of transition-state intermediate	225 s	Confocal	9, 10, 15, 16	[16,21]
MAC formation and cell death	60–240 s	Confocal	8–10, 15	[16,20,21]

* Time estimates are based on a range of experiments and techniques.

**Table 2:** Binding of Al488-labeled C1q to mAb-opsonized Daudi cells and colocalization of C1q with mAb.

	Expt. 1			Expt. 2			Expt. 3 ^a^		
	Al647 mAb (GMF) ^b^	Al488 C1q (GMF)	BDSS ^c^	Al647 mAb (GMF)	Al488 C1q (GMF)	BDSS	Al647 mAb (GMF)	Al488 C1q (GMF)	BDSS
Al647 OFA	181,000	162,000	3.0	116,000	113,000	3.4	206,000	30,000	2.5
Al647 RTX	186,000	7500	0.9	61,000	6600	2.1	155,000	2200	1.0
Al647 7D8 ^d^	133,000	2300	0.6	104,000	1000	0.9	210,000	1300	0.7

^a^ Different Al488 C1q preparation. ^b^ GMF, geometric mean fluorescence. ^c^ BDSS, bright detail similarity score. ^d^ IgG4 (K322A).

## Abbreviations

7AAD	7-aminoactinomycin D
Al	Alexa dye
APC	alternative pathway of complement
BDSS	bright detail similarity score
C	complement
CDC	complement-dependent cytotoxicity
CLL	chronic lymphocytic leukemia
EAC	Ehrlich ascites cell
FcεRI	the high-affinity receptor for the Fc region of IgE
FcγR	receptor for the Fc region of IgG
GMF	geometric mean fluorescence
HRDI	high-resolution digital imaging in a flow cytometry environment
MAC	membrane attack complex
mAb	monoclonal antibody
NHS	normal human serum
OFA	ofatumumab
PI	propidium iodide
RTX	rituximab
TNTs	tunneling nanotubules
TMRME	tetramethylrhodamine methyl ester
